# Effect of switching from prior Nucleos(t)ide Analogue(s) to Tenofovir alafenamide on lipid profile and cardiovascular risk in patients with Chronic Hepatitis B

**DOI:** 10.1371/journal.pone.0324897

**Published:** 2025-05-27

**Authors:** Witchayaporn Praguylertluck, Apichat Kaewdech, Naichaya Chamroonkul, Teerha Piratvisuth, Pimsiri Sripongpun

**Affiliations:** 1 Division of Internal Medicine, Faculty of Medicine, Prince of Songkla University, Hat Yai, Thailand; 2 Gastroenterology and Hepatology Unit, Division of Internal Medicine, Faculty of Medicine, Prince of Songkla University, Hat Yai, Thailand; 3 NKC Institute of Gastroenterology and Hepatology, Songklanagarind Hospital, Prince of Songkla University, Hat Yai, Thailand; Kaohsiung Medical University, TAIWAN

## Abstract

**Introduction:**

Tenofovir alafenamide (TAF) is recommended for chronic hepatitis B (CHB) treatment in international guidelines according to its efficacy and safety. However, in phase III study, an increased LDL-c was observed in those who were switched from Tenofovir disoproxil fumarate (TDF) to TAF. Limited data exists on whether lipid profiles change only in individuals who switched to TAF from TDF or from any nucleoside/nucleotide analogues (NUC). We investigated how switching to TAF affected lipid and cardiovascular outcomes in Thai CHB patients.

**Materials and methods:**

We conducted a prospective observational study including CHB patients who had to switch from their prior NUC to TAF according to the national reimbursement policy in late 2022. All enrolled patients had lipid tests and transient elastography (TE) done at 0 and 48-week post-switch to TAF. Demographic data, prior NUC, liver biochemistry, controlled attenuated parameter (CAP) and liver stiffness (elastic modulus; E) data measured by TE were collected. The changes in lipid, Thai cardiovascular (CV) risk score, and TE results between 0 and 48-week were compared.

**Results:**

A total of 110 patients who were switched to TAF and completed 48-week follow-up were analyzed. The prior NUCs were as follows: 47 Lamivudine (LAM), 22 Entecavir (ETV), and 41 TDF-based. Baseline characteristics were similar between the three groups except for underlying hypertension was more frequent and baseline total cholesterol was lower in the TDF-based group. At 48-week post-switch, the median LDL-c changes were -2.45, -5.9 and +8.8 mg/dL (p<0.001), and total cholesterol changes were -4.5, -4 and +17 mg/dL (p<0.001), in the ETV, LAM, and TDF-based group, respectively. Whereas the changes in hepatic steatosis (measured by CAP), and liver stiffness (measured by E) as well as Thai CV risk score were not significantly different. No cardiovascular events occurred during follow-up.

**Conclusion:**

Significant increase in LDL-c and total cholesterol after switching to TAF were observed only in patients with prior TDF, but not in those with prior ETV or LAM. Careful monitoring of lipids after the switch may not be universally needed. Data regarding long-term cardiovascular outcomes are warrant.

## Introduction

Chronic viral hepatitis B (CHB) infection is a renowned global health issue, causing an estimated 350,000 deaths in 2019, primarily due to cirrhosis and hepatocellular carcinoma (HCC) [1]. Antiviral therapies, including lamivudine (LAM), entecavir (ETV), tenofovir disoproxil fumarate (TDF), and tenofovir alafenamide (TAF), have been proven to control viral load, reduce liver inflammation, delay cirrhosis progression, lower liver cancer incidence, and, therefore, become standard treatment of CHB. Due to the need for long-term treatment, recommendations from international liver societies, especially in Europe and the United States suggested that high genetic barrier agents, namely ETV, TDF, and TAF, should be considered as the first line treatment in all patients [2,3]. More specifically, in patients over 60 years, those with osteoporosis, or impaired kidney function, ETV or TAF are preferred over TDF.

In many resource-limited settings, the high cost of the preferred agents has been a significant barrier to access, limiting their widespread use. In Thailand, for example, LAM remained the first-line treatment for CHB in the National List of Essential Medicines for many years [4]. However, in 2017, ETV was approved as a first-line treatment in patients with advanced fibrosis, cirrhosis, or high baseline HBV DNA levels. Despite this, most CHB patients continued to receive LAM, with TDF added if treatment targets were not met [4,5] or drug resistance developed.

TAF, the latest agent approved for CHB, offers superior renal and bone safety compared to TDF, maintaining high antiviral efficacy by delivering 4–7 times higher concentrations of the active ingredient, tenofovir, in target cells [6–8]. In December 2022, TAF was added to Thailand’s National Essential Medicine List as the preferred first-line therapy for CHB, applicable to all patients aged 18 and older starting treatment, and for those switching from LAM, ETV, or TDF. Although TAF is associated with favorable safety profiles, some studies suggest it may increase total cholesterol and LDL levels [9–11], especially when switching from TDF, which might increase cardiovascular (CV) risk, and it may be linked to greater weight gain. Concerning the changes in lipid profiles after switching from TDF to TAF, data regarding switching from other antiviral agents to TAF are limited. This study investigates the impact of switching from prior antiviral treatments to TAF on lipid profiles, liver fat, and CV risk in CHB patients.

## Materials and methods

We conducted a prospective observational study at Songklanagarind Hospital, a tertiary care hospital in Southern Thailand, including CHB patients aged ≥18 years who were regularly followed up at our center, received the stable nucleos(t)ide analogue(s) (NUC) regimen for at least 3 months, and had to switch from their prior NUC to TAF according to Thailand national reimbursement policy after December 2022. Patients with concomitant active HCC or HIV co-infection were excluded, as well as patients who had to stop TAF after the switch within 1 year and those who successful transient elastography (TE) cannot be performed. The recruitment of study participants started on January 25, 2023 and the last eligible participant was enrolled on July 25, 2023.

### Data collection

Baseline demographic data were collected at the time of switching to TAF (week 0) including age, sex, smoking status, underlying diseases, body mass index (BMI), liver blood test, and data regarding CHB treatment e.g., antiviral regimen, HBV DNA, cirrhosis status. Total cholesterol (TC), and low-density lipoprotein-cholesterol (LDL-c) were also collected at week 0. All eligible patients would undergo transient elastography (TE) using Fibroscan® 502 Touch (Echosens) to evaluate liver fat and liver stiffness at week 0 as well.

Following the switch, eligible patients were monitored by their attending physicians as part of routine CHB management. Lipid profiles, liver blood tests, and TE results were re-evaluated at 48 ± 4 weeks post-switch. Any adjustments to lipid-lowering therapy or treatment for cardiovascular disease were made at the discretion of the attending physician. Data regarding the administration or change of lipid-lowering agents were also recorded.

The study has been approved by the human research ethics committee (HREC) of Faculty of Medicine, Prince of Songkla University (REC.65-512-14-1). Written informed consent was obtained from all enrolled participants. The study was conducted in accordance with the declaration of Helsinki and Good Clinical Practice.

### Operational definitions

The risk of cardiovascular disease was calculated from the Thai CV risk score (https://www.rama.mahidol.ac.th/cardio_vascular_risk/thai_cv_risk_score/), which derived from the cohort of Thai individuals [12], using the following variables: age, sex, diabetes status, smoking status, systolic blood pressure, and TC levels. Liver fat and liver stiffness was evaluated by Fibroscan® using the controlled attenuated parameter (CAP) and elastic modulus (E) values, respectively.

For the change in LDL-c outcome, due to the prospective observational nature of our study, a standardized protocol for managing lipid profile derangements was not established. Decisions regarding the initiation or adjustment of statin therapy were made at the discretion of the primary physicians. Consequently, patients who exhibited elevated levels of LDL-c or TC prior to the 48-week timepoint may have undergone treatment modifications, which potentially resulted in lower lipid levels observed at the 48-week follow-up. Therefore, we combined the increased LDL-c level and the need to initiate or increase statin dose as another outcome, namely ‘worsening LDL-c level’.

### Statistical analysis

Based on the previous study by Ogawa E(10), patients in TDF-based group experienced an increase in LDL-c level 15 ± 18 mg/dL and patients in the other NUC group had an increase in LDL-c level 5 ± 18 mg/dL. A sample size of at least 102 patients was calculated to provide 80% power with a level of significance at 0.05. To account for potential loss to follow-up at week 48, we planned to round up the sample size to 110 patients.

All statistical analyses were conducted in R program version 4.2.2 (Vienna, Austria). Comparisons of continuous variables between week 48 and at baseline in the same individuals were analyzed using paired t-test or Wilcoxon sign-rank test according to the distribution of the data. The comparisons of the outcomes between the prior TDF-based group and other NUC (LAM and ETV) groups were carried out using Chi-square test or Fisher Exact test for categorical variables and ANOVA F-test or Kruskal-Wallis test for continuous variables, as appropriate, using the ∆ (change) in the observed values between baseline (week 0) and at the time of follow up (week 48). To identify potential variables associated with the ‘worsening LDL-c level’ outcome, we performed univariable logistic regression analyses, and all variables with p < 0.2 from univariable analyses will be then entered into the multivariable logistic regression analysis, as well as the adjustment for age, sex, and known potential variables for LDL-c change e.g., change in BMI. A 2-tailed p-value of < 0.05 was considered to be statistically significant.

## Results

**Fig 1:**
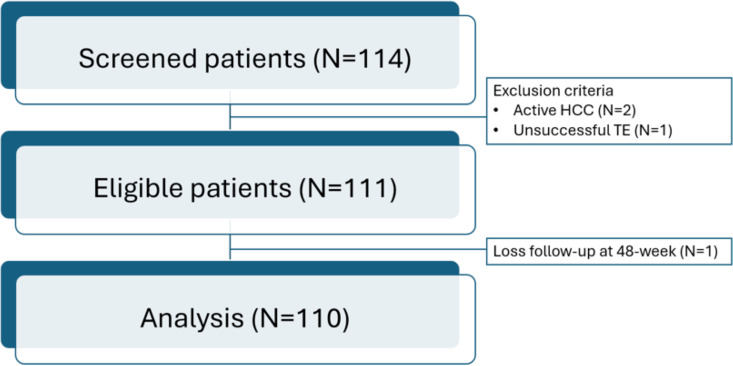
Study flow of study participants.

During the study period, 114 CHB patients who were switched from their prior NUC to TAF were screened; 2 patients had active HCC, 1 was excluded due to unsuccessful TE, and one patient lost to follow-up at week 48 (Fig 1). Thus, the remaining 110 patients were included in the analysis.

### Baseline characteristics

Of the 110 patients, we divided eligible patients into three groups according to the previous antiviral regimen: ETV monotherapy, LAM monotherapy and TDF-based group (including both TDF monotherapy and dual LAM-TDF or ETV-TDF). Baseline characteristics of the patients in the three groups are shown in Table 1. There were no significant differences between the three groups in terms of demographic data, age, sex, BMI, HBV DNA, alanine aminotransferase (ALT), LDL-c, CAP, liver stiffness, cirrhosis status, and underlying diseases except for hypertension, which was more prevalent in the TDF-based group. Interestingly, the serum TC level at the time of switch was lowest in the TDF-based group. The Thai CV risk score was also higher in the TDF-based group than the other 2 groups.

**Table 1:** Baseline characteristics at the time of switch to TAF (N = 110).

	ETV mono (N = 22)	LAM mono (N = 47)	TDF-based (N = 41)	P value
Age, year, mean (SD)	53.05 (11.22)	55.11 (11.92)	59.46 (11.77)	0.080
Male sex, n (%)	15 (68.18)	26 (55.32)	24 (58.54)	0.596
Weight, kg, mean (SD)	65.35 (12.13)	63.14 (10.61)	64.23 (13.46)	0.766
BMI, kg/m2, mean (SD)	24.02 (3.62)	24.26 (3.79)	24.56 (4.37)	0.867
*Underlying disease*				
DM, n (%)	4 (18.18)	5 (10.64)	6 (15)	0.670
HTN, n (%)	5 (22.73)	6 (12.77)	16 (39.02)	0.017
DLP, n (%)	10 (45.46)	13 (27.66)	13 (31.71)	0.335
CKD, n (%)	1 (4.55)	4 (8.51)	4 (9.76)	0.905
CVS, n (%)	0 (0)	3 (6.38)	0 (0)	0.226
Statin use at baseline, n (%)	9 (42.86)	8 (17.02)	12 (30)	0.072
*HBV status*				
HBV DNA, U/L, median (IQR)	0 (0,0)	0 (0,0)	0 (0,0)	0.306
Detectable HBV DNA, n (%)	5 (22.73)	6 (13.33)	3 (7.69)	0.250
Cirrhosis, n (%)	7 (31.82)	21 (44.68)	24 (58.54)	0.115
*Laboratory data*				
AST, U/L, median (IQR)	26.5 (18.25,30.75)	27 (20.5,31.5)	27 (20,32)	0.884
ALT, U/L, median (IQR)	24 (17.25,30.5)	25 (17,32.5)	25 (17,32)	0.971
TC, mg%, mean (SD)	185 (38.49)	188.19 (41.44)	163.63 (26.72)	0.005
LDL, mg%, mean (SD)	119.27 (37.0)	118.76 (30.03)	107.59 (28.90)	0.186
Cr, mg/dL, mean (SD)	0.94 (0.19)	0.88 (0.18)	0.90 (0.19)	0.521
*Transient elastography*				
CAP, dB/m, mean (SD)	206.14 (55.29)	220 (63.88)	230.20 (53.27)	0.298
E, kPa, median (IQR)	7.5 (4.63,10.45)	7.2 (5.55,9.45)	6.4 (4.6,9.1)	0.568
*Thai CV risk*				
Risk score, median (IQR)	6.82 (2.43,17.24)	8.06 (3.98,13.75)	15.81(5.19,24.19)	0.029
CV Risk group				0.021
<10%	14 (63.64)	30 (63.83)	15 (37.5)	
10-20%	5 (22.73)	9 (19.15)	7 (17.5)	
>20%	3 (13.67)	8 (17.02)	18 (45)	

**BMI, body mass index; WC, waist circumference; DM, diabetes mellitus; HTN, hypertension; DLP, dyslipidemia; CKD, chronic kidney disease; CVS, cardiovascular system disease; ALT, alanine transaminase; AST, aspartate transaminase; Cr, creatinine; CAP, controlled attenuation parameter; E, elastic modulus; TC, total cholesterol; LDL, low density lipoprotein

### Post switch 48-week

The characteristics of patients in all 3 groups at 48-week post switch to TAF are shown in Table 2. At 48-week post-switch, all three groups exhibited sustained viral suppression, as undetectable HBV DNA at week 48 were observed in 89% (17/22), 93% (43/47) and 92% (35/41) in the ETV, LAM and TDF-based group, respectively. For the biochemical response, reduction in ALT levels compared to baseline were observed across all 3 groups, but the degree of ALT reduction was not significantly different among groups.

**Table 2:** Outcomes at the time of 48-week post switch (N = 110).

	ETV mono (N = 22)	LAM mono (N = 47)	TDF-based (N = 41)	P-value
Weight, kg, mean (SD)	65.50 (10.01)	63.18(11.07)	65.77 (13.34)	0.547
BMI, kg/m2, mean (SD)	24.12 (2.88)	24.25 (3.86)	25.18 (4.48)	0.459
WC, cm, median (IQR)	88 (82,96)	89.5 (80,96.75)	89 (82,99)	0.828
*Laboratory data*				
HBV DNA, U/L, median (IQR)	0 (0,0)	0 (0,0)	0 (0,0)	0.843
AST, U/L, median (IQR)	28.5 (24,32.75)	28 (24,32.5)	27 (22,31)	0.426
ALT, U/L, median (IQR)	24.5 (16,28.75)	21 (14.5,32)	19 (13,29)	0.745
ALP, U/L, median (IQR)	78 (62,89)	80 (65.5,105)	73 (67,91)	0.882
Total cholesterol, mg%, mean (SD)	175.91 (36.01)	183.43 (42.59)	181.66 (41.33)	0.775
LDL, mg%, mean (SD)	112.71 (34.74)	113.85 (34.66)	119.35 (39.96)	0.716
Cr, mg/dL, mean (SD)	0.94 (0.19)	0.88 (0.18)	0.90 (0.19)	0.521
*Transient elastography*				
CAP, dB/m, mean (SD)	210.68 (55.01)	221.09 (66.95)	227.54 (59.01)	0.588
E, kPa,median(IQR)	5.8 (4.58,8.32)	6.4 (4.4,8.1)	6.3 (4.7,8.8)	0.795
***Changes of Laboratory data and characteristics***				
∆ weight, kg, median(IQR)	0 (-1.3,0.85)	0 (-1,1.75)	1 (0,3)	0.010
∆ BMI, kg/m^2^, median(IQR)	0 (-0.53,0.33)	0 (-0.45,0.64)	0.42 (0,1.28)	0.009
∆ LDL, mg%, median(IQR)	-2.45 (-13.15,10.93)	-5.9 (-16.55,9.9)	8.8 (2,19.8)	< 0.001
∆ CHOL, mg%, median(IQR)	-4.5 (-15.75,6.75)	-4 (-15.5,13)	17 (4,31)	< 0.001
∆ Cr, mg/dL, median(IQR)	0.03 (-0.02,0.07)	0.005 (-0.03,0.06)	0 (-0.03,0.03)	0.245
∆ AST, U/L, median(IQR)	2.5 (-1.75,7.75)	3 (-1.5,7)	1 (-4,6)	0.345
∆ ALT, U/L, mean(SD)	-3 (7.32)	-2.87(10.76)	-4.83(10.28)	0.623
∆ CAP, dB/m, mean(SD)	4.55 (66.27)	1.09 (71.7)	-2.66 (66.00)	0.920
∆ E, kPa, median(IQR)	-0.4 (-2.1,0.15)	-0.3 (-1.8,0.91)	-0.1 (-1.3,1.3)	0.320
Detectable HBV DNA, n (%)	2 (10.53)	3 (6.52)	3 (7.90)	0.90
∆ HBV DNA, U/L, median (IQR)	0 (0,0)	0 (0,0)	0 (0,0)	0.695
*Treatment of dyslipidemia*				
increase dose or potency of statin,n (%)	0 (0)	5 (10.64)	6 (14.63)	0.185
*Thai CV risk*				
Thai CV Risk score, median (IQR)	8.79 (2.4,18.00)	9.41 (3.74,18.05)	13.87 (7.31,24.37)	0.044
Thai CV Risk score change, median (IQR)	0.10 (-0.66,1.52)	0.51 (-0.38,2.70)	0.61 (-0.99,3.76)	0.544
CV risk group				0.069
<10%	13 (59.09)	24 (52.17)	13 (31.71)	
10-20%	6 (27.27)	13 (28.26)	11 (26.83)	
>20%	3 (13.64)	9 (19.57)	17 (41.46)	
CV risk group change				0.572
Decrease	1 (4.55)	1 (2.17)	4 (10)	
Same	19 (86.36)	38 (82.61)	32 (80)	
Increase	2 (9.09)	7 (15.22)	4 (10)	

**BMI, body mass index; WC, waist circumference; DM, diabetes mellitus; HTN, hypertension; DLP, dyslipidemia; CKD, chronic kidney disease; CVS, cardiovascular system disease; CVD, cerebrovascular disease; ALT, alanine transaminase; AST, aspartate transaminase; Cr, creatinine; CAP, controlled attenuation parameter; E, elastic modulus; LDL, low density lipoprotein

**Fig 2:**
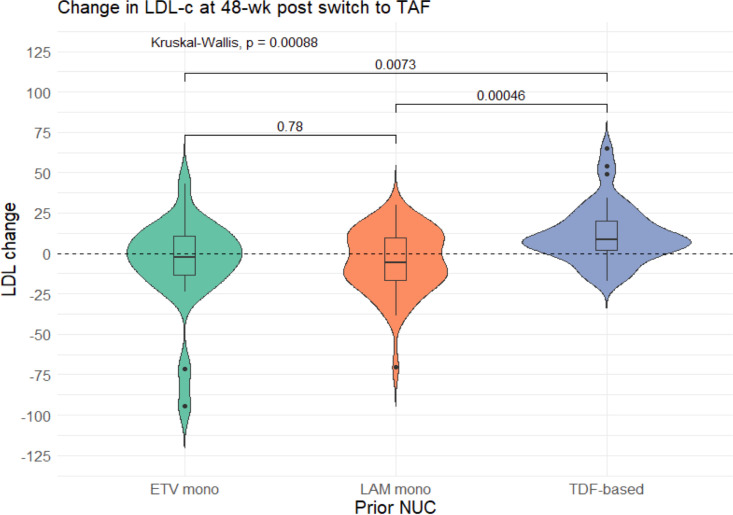
Violin plot of the change in LDL-c levels at 48 weeks post switch.

**Fig 3:**
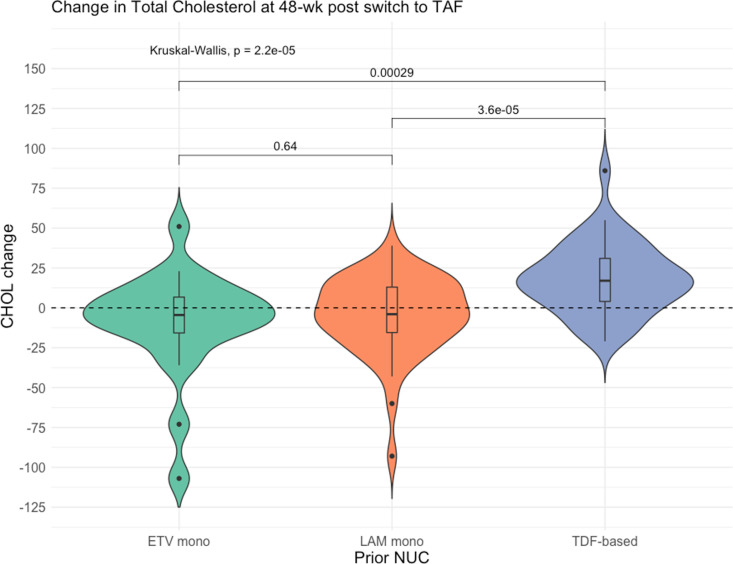
Violin plot of the change in TC levels at 48 weeks post switch.

The median LDL-c changes (Table 2 and Fig 2) between week 48 compared to baseline were -2.45, -5.9, and +8.8 (p < 0.001), and the median total cholesterol level changes (Table 2 and Fig 3) were -4.5, -4, and +17 (p < 0.001) in the ETV, LAM, and TDF-based group, respectively. Moreover, a significant increase in weight and BMI were also observed in the TDF-based group than others. Whereas the changes in waist circumference, CAP, liver stiffness were not significantly different among the three groups.

No new cardiovascular event occurred during follow-up and the Thai CV risk scores were insignificantly changed between all three groups. The modification of statin either increase dosage/ potency/ or new initiation of statin were observed more commonly in the TDF-based group, albeit insignificantly.

### Variables associated with worsening LDL-c level

As noted earlier, because our study was a prospective observational design, the decisions regarding lipid-lowering treatment were made based on the discretion of the attending physicians. As a result, patients who had elevated LDL-c or TC levels before week 48 may have had their treatment modification earlier, which potentially resulted in lower LDL-c levels observed at week 48. To address this, we defined the “worsening LDL-C level” as either an increase in LDL-C levels or the initiation or dose escalation of lipid-lowering therapy before week 48 and used this outcome in our logistic regression analyses.

The results of univariable logistic regression analyses determining factors associated with the worsening LDL-c outcome are shown in S1 Table. The results revealed that the only factor significantly associated with worsening LDL-C after switching to TAF was the prior use of a TDF-based regimen. In a multivariable logistic regression analysis, we also entered additional following variables: age, sex, hypertension status, baseline BMI, and change in BMI (as the latter three were more prevalent in TDF-based group compared to others), to assess whether TDF-based regimen was independently associated with the worsening LDL-c. The multivariable analysis confirmed that a TDF-based regimen at baseline, prior to the switch to TAF, was significantly associated with worsening LDL-C, even after adjusting for the aforementioned variables (Table 3).

**Table 3:** Multivariable logistic regression analysis of the variables associated with the worsening LDL-c outcome (N = 110).

Variables	Adjusted OR	95% lower	95% upper	P-value
BMI at day of switch	0.98	0.88	1.09	0.709
∆ BMI	1.06	0.83	1.42	0.629
TDF-based group	3.84	1.55	10.22	0.005
Age	0.10	0.96	1.03	0.83
Male sex	0.75	0.31	1.78	0.518
Hypertension	1.04	0.38	2.90	0.941

**Worsening LDL-c; increase of LDL-c level or increase dose/intensity of statin drug**.

### Sensitivity analyses

Additionally, to further account for the lipid-lowering agent use, we performed a sensitivity analysis in patients who did not receive any lipid lowering medication for the entire 48-week. Of the patients in the cohort, 72 patients who were naïve for lipid lowering therapy were included in this sensitivity analysis. The results are in accordant to the total patients (S2 Table) that the increase in LDL-c and TC levels were higher in the TDF-based group, significantly different from other NUC group.

**Fig 4:**
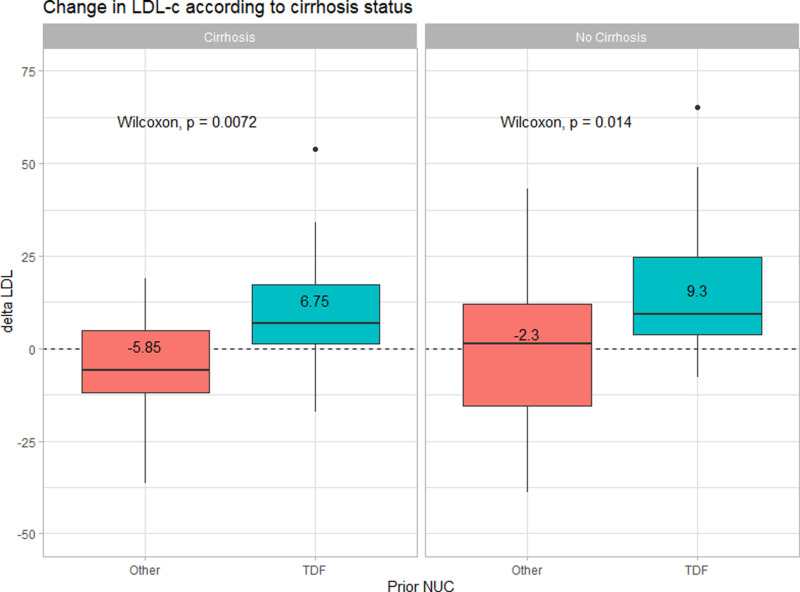
Changes in LDL-c according to cirrhosis status of the patients in the entire cohort (N = 110).

**Fig 5:**
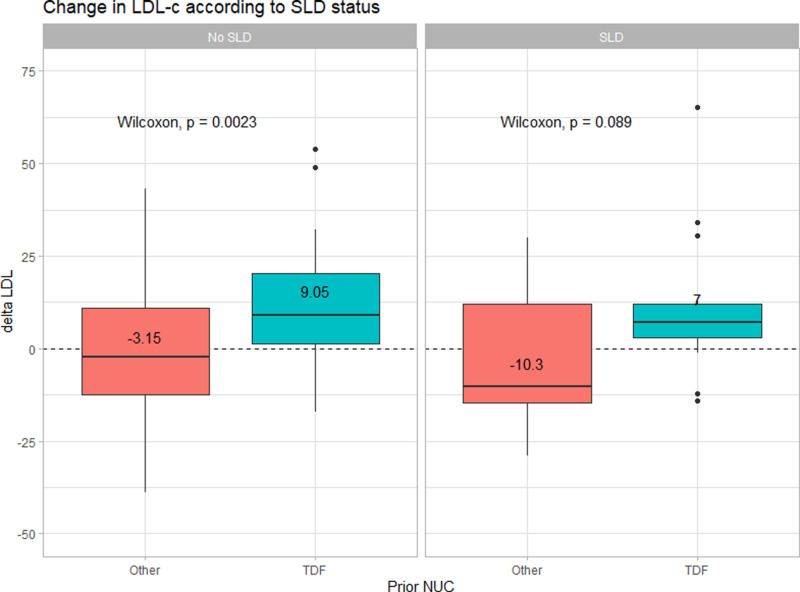
Changes in LDL-c according to SLD status of the patients in the entire cohort (N = 110).

Furthermore, we performed sensitivity analyses by stratifying patients according to their cirrhotic status and co-existing steatotic liver disease (SLD) status to evaluate the changes in LDL-c in those groups of patients as cirrhosis and SLD might have an effect on lipid metabolism. The changes in LDL-c in the TDF-based group were consistently higher than those in other NUC group both in patients with cirrhosis (N = 52) and non-cirrhosis (N = 58) (+6.75 vs -5.85 mg%, p = 0.007 and +9.3 vs -2.3 mg%, p = 0.014, in patients with and without cirrhosis, respectively) as shown in Fig 4. For the SLD status, Fig 5 illustrates the LDL-c changes between those with and without co-existing SLD (defined by CAP > 248 dB/m at baseline). The similar pattern was observed that those with TDF-based group experienced an increase in LDL-c higher than patients who were on other NUC prior to switching to TAF; + 9.05 vs -3.15 mg% (p = 0.002) in the no SLD subgroup (N = 78), and +7 vs -10.3 mg% (p = 0.089) in SLD subgroup (N = 32), respectively.

## Discussion

Our study found that an increase in LDL-c level after switching from prior NUC to TAF in patients with CHB were not universal. Significant increases in LDL-c and TC levels were observed only in patients who were switched from TDF, whereas the lipid levels seemed to be lower in those who were switched from LAM and ETV. However, the CV risk of CHB patients in all 3 groups were not significantly changed.

ETV and TDF had been generally recommended as the first line antiviral agents for the treatment of CHB worldwide as they owe high potency property as well as very low risk of developing drug resistance in the long-term [2,3,13]. Additionally, some studies suggest that TDF might be associated with a lower risk of HCC compared to ETV [14]. Nonetheless, the major drawbacks of TDF are long-term renal and bone toxicities. TAF is the latest approved medication for the treatment of CHB in which posed the advantage of long-term renal and bone safety while maintaining the high efficacy and high genetic barrier of tenofovir prodrug [2,3].

TAF seemed to be the promising antiviral agent in the treatment of CHB. However, the early studies in patients who were switched from TDF to TAF, either patients living with HIV [15] or CHB [16] found that the LDL-c levels significantly increased after the switch. This raised concern regarding the risk of developing cardiovascular events if TAF is widely used in the future [17,18], especially in patients living with HIV as they are at risk of metabolic dysfunction. In patients with CHB, although the metabolic risk was of less concern than those with HIV, the current era of obesity pandemic and coexisting steatotic liver disease [19–22] might have an impact on CV risk of patients with CHB as well.

The result of our study aligns with the prior studies in patients with CHB who were switched from TDF to TAF in which increases in LDL-c, TC levels, and weight gain were observed. In the first randomized controlled trial comparing the switch to TAF versus continuation of TDF in virologically suppressed CHB patients, 4% of the TAF group and 2% of the TDF group reported to have LDL-c > 190 mg/dL [16]. Subsequently, Suzuki K et al also reported that LDL-c and TC levels were significantly increased in patients who were switched from TDF to TAF for 6–12 months in a multicenter retrospective study, and the proportions of patients diagnosed with dyslipidemia also increased from 33% to 39% [9]. Additionally, Ogawa E et al also reported that switching from TDF to TAF resulted in a significant increase in both LDL-c and TC levels at 144 weeks post-switch compared to patients switched from ETV to TAF [10].

Ogawa’s study prompted the consideration that switching to TAF may not necessarily have a negative impact on serum lipid levels in all patients with CHB. Our results support this notion, showing that individuals who transitioned from ETV or LAM monotherapy to TAF did not experience significant changes in their lipid profiles. In contrast, only those previously treated with TDF demonstrated significant alterations, with the prior TDF-based regimen remaining notably associated with worsening LDL-c levels, even after adjustments for weight changes. To date, we have found only one article similar to our study, Cheng et al [23] studied in CHB patients who were on TDF or ETV and switched to TAF and found that the transition from TDF to TAF resulted in significant increases in body weight, total cholesterol, and LDL-c. Conversely, the switch from ETV to TAF did not produce any significant changes in these parameters. The results were concordant with our study.

One potential explanation for this phenomenon is that the issue may not stem from TAF adversely affecting lipid profiles; rather, it is the lipid-lowering effect of TDF that plays a significant role [24–26]. When TDF is discontinued, the previously suppressed lipid levels in these patients are revealed, leading to an increase in LDL-c and TC following the transition to TAF. This rationale is also supported by the evidence that patients who were on TAF as the first-line treatment in CHB did not have a significant lipid alteration [27]. And our data also showed that, at baseline prior to the switch, those who were treated with TDF had significantly lower TC levels compared to the other two groups.

Nonetheless, no new cardiovascular event occurred in all patients in our study. And the lipid alteration in the TDF-based group did not have a significant effect on the CV risk of the patients. Change in body weight is again in accordant with prior studies that those who were switched from TDF to TAF experienced weight gain [23,28], although the mechanism associated with this finding is still unknown. We also evaluated the effect of switching to TAF on liver fat using CAP values measured by TE, there were no differences in changes of liver fat between the three groups despite the difference in changes of serum lipid profiles and body weight.

The strength of our study is that it is a prospective study, outcomes of interest were measured at the specific timeframe systematically. And we presented not only the changes in lipid profiles but also the CV risk and evaluated the CV event. Moreover, to the best of our knowledge, this is the first study evaluating the change in liver fat in accordance with serum lipids in CHB patients who were switched to TAF.

We also acknowledge the limitation of the current study. As it is an observational study, there was no specific protocol for the management of lipid alteration in our cohort, the change or initiation of lipid lowering agent to the patients was at the discretion of their primary attending doctors. However, we have analyzed both changes in the serum lipids as well as the changes in lipid-lowering agents to provide the whole picture of lipid management in our cohort. We also performed a sensitivity analysis in those not receiving lipid lowering therapy during the study period and the results are parallel. It is also noted that, to evaluate the change in lipid profiles in those who were switched to TAF, having the control group of those who continued the same antiviral agent during the same timeframe would be better for comparison. Nonetheless, as the switching to TAF was mandatory according to the Thailand’s reimbursement policy, this precluded us from having such control group. Additionally, the observation period of this study may be relatively short for evaluating the development of cardiovascular event, we used Thai CV risk scores to be the surrogate markers for the long-term outcomes, nevertheless, studies with a longer follow-up time are still needed.

In conclusion, among CHB patients switched from their prior NUC to TAF, significant increases in LDL-c and TC levels were observed only in those who were on TDF at baseline, but not in those previously treated with ETV or LAM. Careful monitoring of lipid levels and timely management of dyslipidemia are recommended for patients who were on TDF prior to the switch to minimize the risk of cardiovascular events. In contrast, patients on baseline ETV or LAM monotherapy were at low risk for lipid alterations following the switch.

## Supporting information

S1 Table Univariable logistic regression analysis for variables associated with the worsening LDL-c outcome. (PDF)

S2 Table Sensitivity analysis in patients not receiving lipid lowering agent during the study period. (N = 72).
(PDF)
