# Supplementary material for: Effect of switching from prior Nucleos(t)ide Analogue(s) to Tenofovir alafenamide on lipid profile and cardiovascular risk in patients with Chronic Hepatitis B
Source: PLoS One. 2025 May 27;20(5):e0324897. doi: 10.1371/journal.pone.0324897 (PMC12112372; doi:10.1371/journal.pone.0324897)
Supplement: S1 Table — (PDF) [file pone.0324897.s001.pdf]

S1 Table. **Univariable logistic regression analysis for variables associated with the worsening LDL-c outcome.**

Variables	Crude OR	95% lower	95% upper	P-value
Age	1.01	0.98	1.04	0.499
Male sex	0.80	0.37	1.75	0.578
Hypertension	1.53	0.62	3.81	0.353
Diabetes	1.42	0.45	4.48	0.546
CKD	0.85	0.22	3.37	0.823
BMI at day of switch	0.99	0.90	1.09	0.816
∆ BMI	1.16	0.88	1.54	0.244
TDF-based group	3.88	1.61	9.33	0.001
Cirrhosis at baseline	1.21	0.57	2.6	0.621
