# Supplementary material for: Effect of switching from prior Nucleos(t)ide Analogue(s) to Tenofovir alafenamide on lipid profile and cardiovascular risk in patients with Chronic Hepatitis B
Source: PLoS One. 2025 May 27;20(5):e0324897. doi: 10.1371/journal.pone.0324897 (PMC12112372; doi:10.1371/journal.pone.0324897)
Supplement: S2 Table — (N = 72). (PDF) [file pone.0324897.s002.pdf]

<u>S2 Table.</u> Sensitivity analysis in patients not receiving lipid lowering agent during the study period. (N=72)

	**Other NUC group (N=47)**	**TDF-based group (N=25)**	**P-value**
Δ weight,kg, median(IQR)	-0.5 (-1.3,1)	1.5 (0, 3.2)	0.002
Δ BMI,kg/m^2, mean±SD	-0.204±1.108	0.737+1.1	<0.001
Δ LDL-c, mg%, median(IQR)	-3.7 (-14.25,11.4)	7.8 (4.6,19.8)	0.002
Δ CHOL,mg%, median(IQR)	-1 (-15,11)	18 (2,28)	< 0.001
Δ Cr, mg/dL, median(IQR)	0.01 (-0.03,0.06)	0 (-0.03,0.03)	0.448
Δ AST, U/L, median(IQR)	4 (-1,8)	1 (-6,6)	0.183
Δ ALT, U/L, mean±SD	-2.553±8.794	-5.64±11.169	0.202
Δ CAP, dB/m, mean±SD	4.021±67.351	-11.44±66.427	0.355
Δ E ,kPa, median(IQR)	-0.2 (-1.75,0.855)	0.1 (-1.3,1.7)	0.381
